# A cost and efficacy analysis of performing arthroscopic excision of wrist ganglions under wide-awake anaesthesia versus general anaesthesia

**DOI:** 10.1186/s12891-020-03482-0

**Published:** 2020-07-13

**Authors:** Cheng-Yo Yen, Ching-Hou Ma, Chin-Hsien Wu, Shih-Chieh Yang, I-Ming Jou, Yuan-Kun Tu

**Affiliations:** 1 Department of Orthopedics, E-Da Cancer Hospital, Kaohsiung, Taiwan; 2 School of Medicine, College of Medicine, I-Shou University, No.1, E-Da Road, Yan-Chau District, 824 Kaohsiung City, Taiwan; 3 Department of Orthopedics, E-Da Hospital, No.1, E-Da Road, Yan-Chau District, 824 Kaohsiung City, Taiwan

**Keywords:** Cost-effectiveness, Efficacy, Ganglion, Wide-awake arthroscopy

## Abstract

**Background:**

Arthroscopic excision has currently become popular for the treatment of wrist ganglions. The objective of this study was to evaluate the clinical outcomes and cost effectiveness of arthroscopic wrist ganglion excisions under Wide-Awake Local Anaesthesia No Tourniquet versus general anaesthesia.

**Methods:**

We retrospectively reviewed patients who underwent arthroscopic ganglionectomy from April 2009 to October 2016 at our institute. They were separated into two groups according to anaesthesia techniques: general anaesthesia and Wide-Awake Local Anaesthesia No Tourniquet. We compared the clinical outcomes and cost-effectiveness of the two groups.

**Results:**

Seventy-four patients were included. Both groups were matched with regard to the demographics and preoperative clinical assessments. We found no significant differences between groups in postoperative visual analog scale, modified Mayo wrist score, Disabilities of Arm, Shoulder and Hand score, recurrence, residual pain, or complications. Recurrence was found in five of 74 patients, one (4.3%) in the Wide-Awake Local Anaesthesia No Tourniquet group and four (7.8%) in the general anaesthesia group. One extensor tendon injury and four extensor tenosynovitis cases occurred in the general anaesthesia group. Regarding cost effectiveness, the mean operating time in the Wide-Awake Local Anaesthesia No Tourniquet and general anaesthesia groups were 88.7 ± 24.51 and 121.5 ± 25.75 min, respectively (*p* < 0.001). The average total costs of the Wide-Awake Local Anaesthesia No Tourniquet and general anaesthesia groups were €487.4 ± 89.15 and €878.7 ± 182.13, respectively (*p* < 0.001).

**Conclusions:**

For arthroscopic wrist ganglion resections, both anaesthesia techniques were effective and safe regarding recurrence rates, complications, and residual pain. The most important finding of this study was that arthroscopic ganglionectomy under Wide-Awake Local Anaesthesia No Tourniquet was superior to that under general anaesthesia for cost-effectiveness.

**Level of evidence:**

Level III, Retrospective comparative study.

## Background

The most common soft-tissue tumours in the hand are ganglions [1]. The aetiology of ganglions could be mucinous degeneration, trauma, synovial herniation, or a one-way valve mechanism, so the precise pathogenesis of ganglions remains unknown [2]. Although the majority of patients experience vague aches, patients tend to seek treatment when their ganglions interfere with activities due to pain or enlargement [2]. Currently, open surgical excision is the mainstay of treatment for wrist ganglions [3] and arthroscopic excision has been described as a favorable alternative [4].

Lalonde et al. [5] has introduced Wide-Awake Local Anesthesia No Tourniquet (WALANT) hand surgery. Several studies have approved the safety and the cost-effectiveness of WALANT [5, 6]. It has been widely used in flexor tendon repairs and other common hand procedures, such as carpal tunnel release, trigger finger release, flexor tendon ganglion excision, hand fractures, and Dupuytren contracture [5, 7]. Few studies have verified the feasibility of WALANT in wrist arthroscopy [8, 9]. One study of WALANT in wrist arthroscopy was published by Hagert and Lalonde [8], in which they reported the clinical outcomes and cost-effectiveness of wide-awake wrist arthroscopy. However, no functional outcomes had been assessed. The authors only declared no adverse events occurred perioperatively. The other study concluded that the application of WALANT wrist and small joint arthroscopy reinforces confidence in surgeons and encourages patients to comply with postoperative rehabilitation. Again, they did not report the functional results of WALANT arthroscopy.

When patients go to the clinic with ganglions most of them seek treatment to excise the lump. There is a need to know whether the introduction of WALANT in arthroscopic ganglionectomy will decrease the recurrence rate and achieve superior clinical outcomes. This study aimed to evaluate the clinical outcomes and cost-effectiveness of arthroscopic wrist ganglion excisions under WALANT. Therefore, we hypothesized that: (1) arthroscopic ganglionectomies under WALANT could achieve comparable clinical outcomes to those performed under general anesthesia (GA), and (2) the operating times and total costs could be significantly reduced.

## Methods

### Study design

We reviewed 84 consecutive patients who underwent arthroscopic ganglionectomy at our institute from April 2009 to October 2016. We had explained the pros and cons of WALANT and GA and then let the patient make a choice. They were separated into two treatment groups according to the patient’s decision in this retrospectively analysis. One of the groups was treated using the WALANT technique and the other under GA. This study had been conducted in accordance with Declaration of Helsinki and was approved by the institutional review board of our hospital on Mar 30, 2018 (EMRP-107-035). We established the diagnosis of ganglion, once a physical examination identified a lump in the wrist, and ultrasonography, or magnetic resonance image (MRI) demonstrated a ganglion sac. Patients with pre-existing interosseous ligament injury, arthropathy, and previous fracture history were excluded. All enrolled patients had been followed for more than 24 months. The authors collected patient demographic data, medical histories, dates of surgery, durations of follow-up, and perioperative complications by reviewing medical records. Preoperative clinical conditions were assessed by calculating visual analog scale (VAS), Disabilities of the Arm, Shoulder and Hand Outcome Measure (DASH), and modified Mayo wrist scores.

### Anaesthesia

#### WALANT group

The key to successful wide-awake surgery depends on the combination of: (1) using lidocaine with epinephrine and (2) sufficient time to allow the anaesthesia to take effect prior to surgery. Therefore, all injections of lidocaine with epinephrine were administered at least 30 min before surgery [8]. Before the surgery we had explained to patients that the operation would be rescheduled and performed under general anaesthesia if WALANT did not work well. We prepared 20 mL of 1% lidocaine with epinephrine 1:100000 and used 27-gauge needles for the initial injections. This kind of tumescent local anaesthesia functions just like a tourniquet-free extravascular Bier block, in which lidocaine with epinephrine is injected subcutaneously only where it is needed [10]. We injected approximately 2 mL of lidocaine with epinephrine into the wrist subcutaneous tissues around each portal, which was established during surgery. Consequently, the radiocarpal and midcarpal joints were injected with 5 mL lidocaine and epinephrine. According to the literature, diluted epinephrine showed potential benefits by reducing intra-articular bleeding and improved surgeon-rated visualization [11]. During the injections, we educated the patients on postoperative care. We injected patients on stretchers outside the minor procedure room and let them wait for the blocks to work. All arthroscopic ganglionectomies were performed in minor procedure rooms without monitored anaesthesia care, sedation, or anaesthesia personnel.

#### GA group

Standard general anaesthesia was administered in this group. Monitored anaesthesia care, sedation, and anesthesia personnel were all involved in these surgeries. In this group, arthroscopic ganglionectomies were done in the operating room. The key difference in each setting was that in the minor procedure room, the surgeon administered anaesthetic, whereas in the operating room, there was an anaesthesiologist administering the anaesthetic. We performed the same intra-articular injections with lidocaine and epinephrine. After the surgery, the patients were transferred to the recovery room for further monitoring.

### Surgical technique

**Fig. 1:**
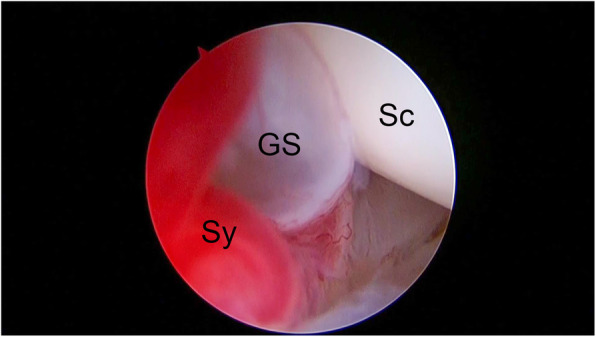
A ganglion stalk. GS, ganglion stalk; Sc, scaphoid; Sy, synovitis

**Fig. 2:**
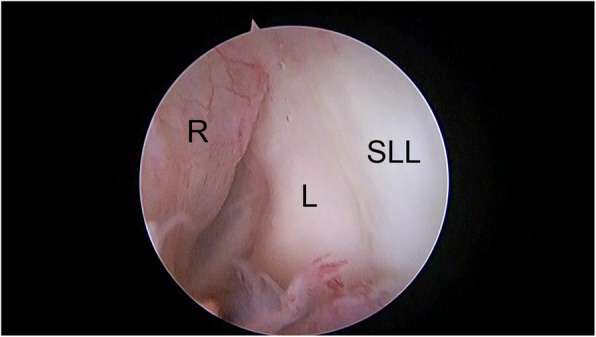
Redundant capsular tissue with synovitis. L, lunate; R, redundant capsular tissue; SLL, scapholunate ligament

After either GA or WALANT was administered smoothly, a vertical traction tower was prepared to suspend the patient’s arm with a 10 ~ 12 lb. traction. Although we never inflated a tourniquet, it was still applied in case visualization was obscured by intra-articular bleeding. A 2.4-mm 30° angulated arthroscope was adopted for the procedure. For dorsal ganglions, a 6-R portal was established as the visualization portal, and a 3–4 portal was used for volar ganglions. Initially, a systematic and thorough examination of the radiocarpal and midcarpal joints was undertaken following a sequence from volar to dorsal, and radial to ulnar. We specifically focused on the condition of the radiocarpal ligaments, the joint capsule, the scapholunate (SL) ligament, the lunotriquetral (LT) ligament, and the triangular fibrocartilage complex (TFCC). The presence of a discrete ganglion stalk (Fig. 1), an intra-ligamentous stalk, or redundant capsular tissue with synovitis (Fig. 2) were documented [12]. We classified the TFCC lesions according to the Palmer criteria. The Geissler arthroscopic classification system was adopted to evaluate the integrity of the interosseous ligaments, such as SL and LT ligaments. All concomitant intra-articular pathologies were treated simultaneously. For dorsal ganglions, the stalk or hyperplasia synovitis was commonly found around the junction of the SL ligament and dorsal capsule. Regarding the volar ganglion, the stalk or synovitis presented in the interval of the volar radiocarpal ligaments. The 1–2 and 3–4 portals were established as the working portal to resect the lesions of volar and dorsal ganglions, respectively. Some cases required the additional radial midcarpal portal to resect the dorsal ganglion completely. Care was taken to preserve the surrounding tendons and ligaments. The decompression of the ganglion cyst would be confirmed by the intraarticular extrusion of gelatinous material. However, the end of resection would be about 1-cm^2^ of the capsule with lesion had been excised. Then, palpation of the previous lump location was recommended to approve the efficacy of the resection [13].

### Postoperative assessment

To determine efficiency and cost effectiveness between the WALANT and GA groups, the operating times and the total costs were reviewed. The total cost was defined as the reimbursement from the government health insurance system. In our system, surgical fees should contain equipment for the operation including local anaesthesia and surgical tools. Basically, if the surgeons administered anaesthetic, they cannot reimburse anaesthesia fees. Therefore, in the WALANT group the cost consisted of the surgical fee only. In the GA group, the cost was the summation of the surgical fee, the anaesthesia fee and the admission fee. The operating time was the time needed to perform the arthroscopic ganglionectomy, set up, and turn over the room. In our country, all GA or brachial plexus block should be administered in the operation room under monitoring. We cannot proceed to next surgery once patient still stay in the operation room. Therefore, the operating time was the summation of surgical and anaesthesia time in GA group. In WALANT group, the operating time consisted of the time to perform the arthroscopic ganglionectomy only. From an economic standpoint, time saving could allow for a greater number of surgeries in a given time. It meant that the surgery was done in an efficient way.

All enrolled patients had been followed for more than 24 months. Clinical outcomes such as residual pain, functional results, recurrence, and complications were assessed at each follow-up visit to gauge the efficacy. The definition of residual pain was a VAS score of 2 or worse. At the final follow-up visit, the functional results were evaluated on the basis of VAS, DASH, and modified Mayo wrist scores. Reappearance of a lump at the same location was defined as a recurrence. Complications like hematoma, wound infection, nerve, vascular, or tendon injuries were recorded. We also recorded adverse events related to epinephrine injections.

### Statistics

The target sample size was projected on the basis of the rates of recurrence [14]. We calculated it using an a priori power analysis. At our institute, arthroscopic excisions had a recurrence rate of 4%. However, some studies reported a recurrence rate as high as 11 to 40% [15]. We chose the average value for the higher recurrence rate, at 25.5%. Thus, this study was designed to have an 80% power of detecting a difference of 20% between the recurrence rates of the two groups. A power analysis with α = 0.05 determined that a sample size of 84 patients was needed.

The SPSS statistical software package (SPSS Inc., Illinois, USA) was adopted to analyse data. An independent *t*-test was used for comparisons of continuous data (age, duration of cyst existence, operating time, total costs, and durations of follow-up). The Mann-Whitney U test was applied for ranked continuous data such as VAS, DASH and modified Mayo wrist scores. The Fisher’s test was used for categorical data (sex, recurrent ganglions, injured side, coexisting pathology, recurrence, residual pain, and complications). The tests were 2-tailed, and *p* < 0.05 was considered statistically significant. Furthermore, we analysed the confounding factors of the operating time or cost using a regression model.

## Results

During the follow-up, one patient presented with scaphoid non-union, one patient subsequently got a clavicular fracture (both in GA group), and eight were lost to follow-up (six in GA group, two in WALANT group). Thus, 74 patients were included in the study. Twenty-three ganglions were resected using WALANT and the remaining 51 ganglions were excised under GA. No surgery was postponed or rescheduled due to WALANT failing. The mean follow-up was 53.3 ± 20.26 months. All the operations were performed by a single surgeon. The numbers of demographic data showed no significant differences between the groups (Table 1). There were 51 scapholunate injuries and 30 TFCC injuries in 64 patients (45 in GA group, 19 in the WALANT group).

**Table 1:** Demographic Data of the Patients

	GA (***N*** = 51)	WALANT (***N*** = 23)	***p*** Value
Age (years)	32.6 ± 9.88	34.6 ± 10.59	0.442
Sex (female)	32 (62.7%)	13(56.5%)	0.618
Injured side (Dominant)	31 (60.8%)	13 (56.5%)	0.801
Duration (months)	15.3 ± 15.00	17.8 ± 11.37	0.478
Recurrent ganglions	17 (33.3%)	6(26.1%)	0.597
Preop VAS	4 (2 ~ 8)	3 (2 ~ 6)	0.064
Preop Mayo	60 (35 ~ 85)	70 (40 ~ 85)	0.559
Preop DASH	31.8 (9.1 ~ 72.7)	25(11.4 ~ 56.8)	0.219
Follow-up (months)	53.8 ± 20.98	50.3 ± 18.36	0.491

Age, duration, and follow-up were analyzed using an independent t-test. Fisher’s tests were used to calculate the difference in the rates of gender, injured side, and recurrent ganglions. The preop VAS, Mayo, and DASH scores were analyzed using Mann-Whitney U tests

*GA* General anesthesia technique, *WALANT* Wide-awake local anesthesia no tourniquet technique, *VAS* Visual analog scale, *Mayo* Modified Mayo wrist scores, *DASH* Disabilities of Arm, Shoulder and Hand Outcome Measure

### Clinical outcomes

#### Recurrence rates

All the recurrences occurred in female patients. In the WALANT group, one patient had a recurrence. Four recurrences were identified in the GA group, which did not represent a significant difference.

#### Functional results and residual pain

The VAS, DASH, and modified Mayo wrist score questionnaires were administered at the final follow-up. There were no significant differences between the groups in the VAS, DASH, and modified Mayo wrist scores. The median VAS scores of the GA group and the WALANT group were 0 (0 ~ 3) and 0 (0 ~ 3), respectively. Therefore, two patients in the WALANT group and six in the GA group had residual pain.

#### Complications

No major medical complications occurred perioperatively. There were no adverse events such as tissue necrosis, shakes or vasovagal fainting. However, while resecting the dorsal capsule we caused one episode of extensor tendon injury in the GA group. Following simultaneous type IB TFCC repair, three cases of ulnar-sided wrist pain were noted (one in GA, two in WALANT). Six months later, those patients did not have residual pain after the suture material was absorbed. There were four cases of extensor tenosynovitis and four cases of wrist stiffness in the GA group. No cases of extensor tenosynovitis were identified at 3 months follow-up. The number of complications in the GA group and the WALANT group were ten and two, respectively. Patients in the GA group tended to have a higher rate of extensor tendon problems, although the number of patients did not achieve a significant difference.

### Efficiency and cost-effectiveness

**Fig. 3:**
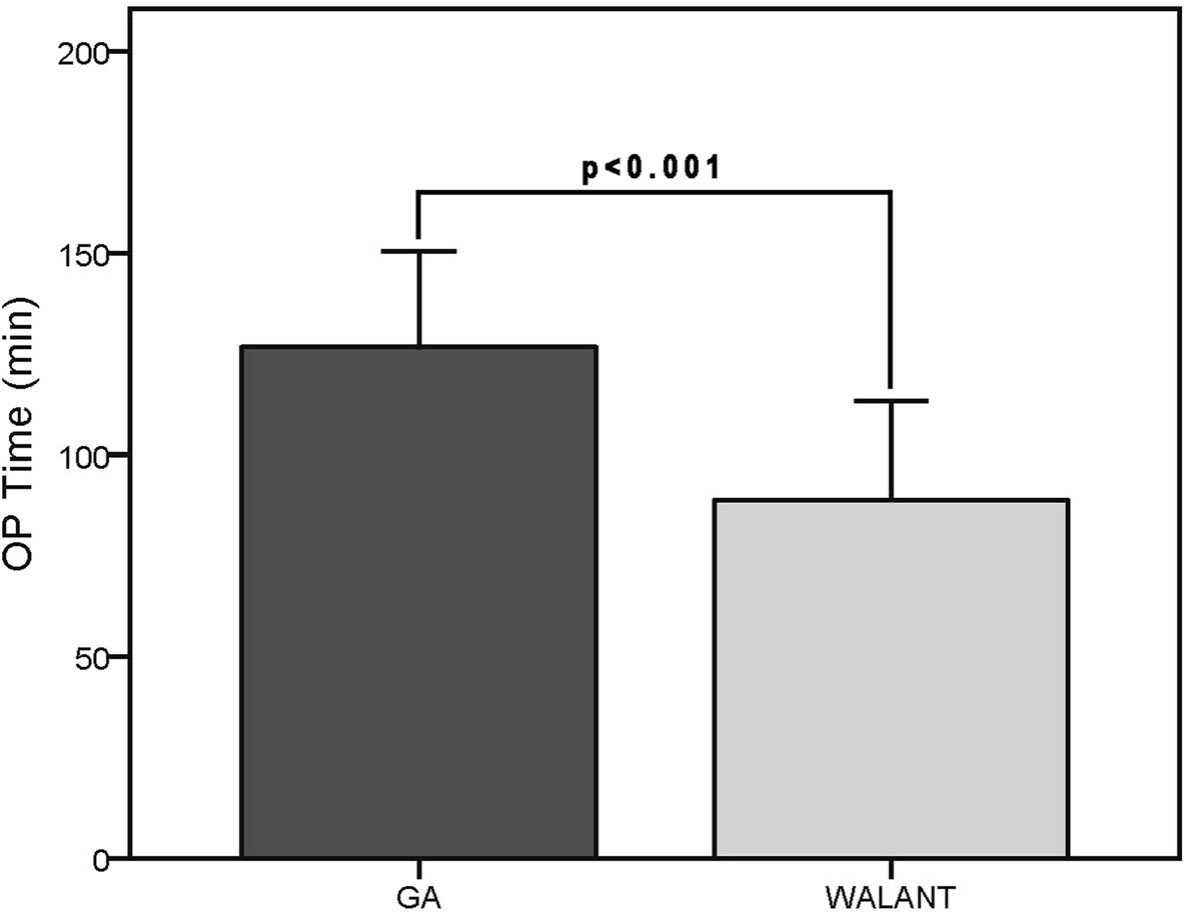
The mean operating time (OP time) in the WALANT and GA groups. Whiskers indicate the standard deviation. Min, minutes

**Fig. 4:**
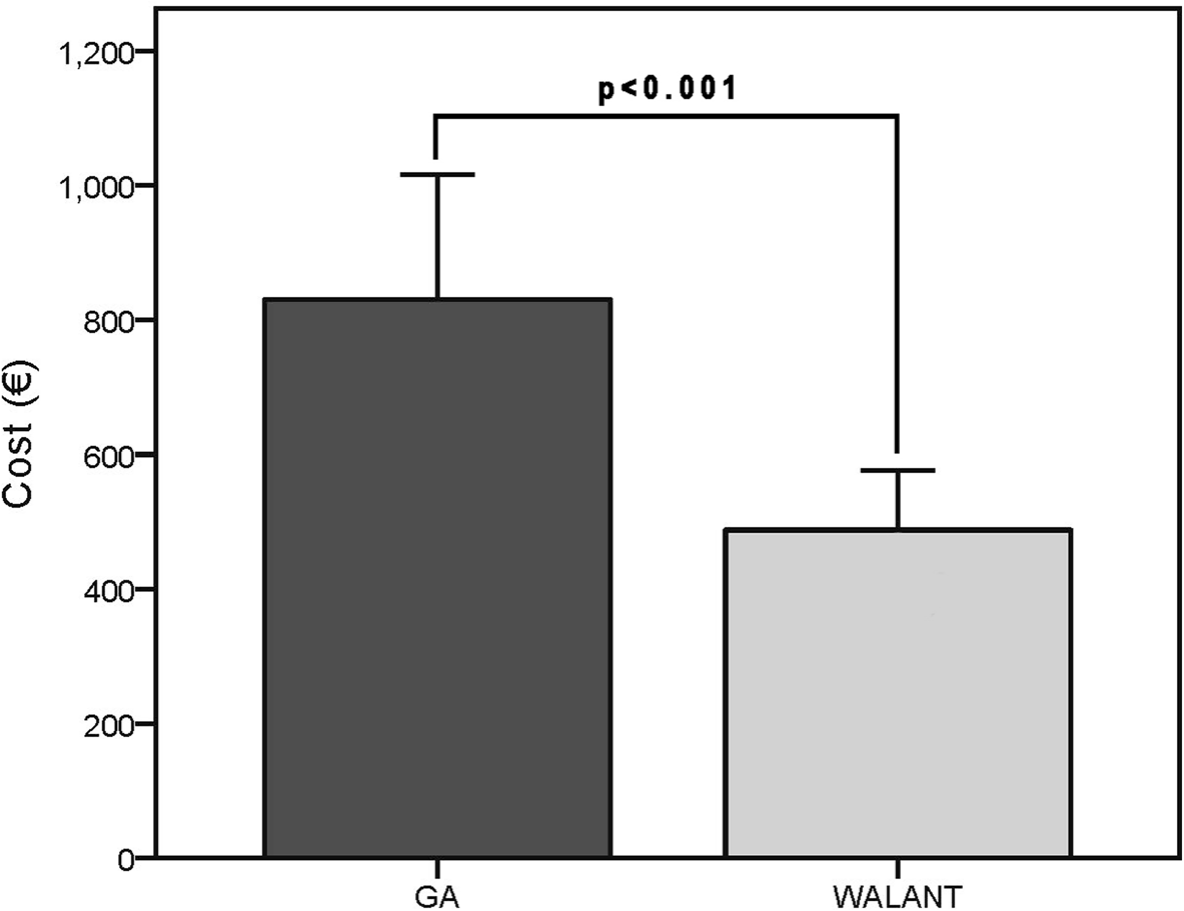
The mean cost in the WALANT and GA groups. Whiskers indicate the standard deviation. €, euro

Regarding efficiency, the mean operating time in the WALANT and GA groups were 88.7 ± 24.51 and 121.5 ± 25.75 min, respectively (Fig. 3). The average total costs of the WALANT and GA groups were €487.4 ± 89.15 and €878.7 ± 182.13, respectively (Fig. 4). The differences in clinical outcomes, efficiency, and cost-effectiveness between groups are summarized in Table 2. The breakdown of the total costs was recorded in Table 3. In the regression analysis, we found that the operating time could be predicted by anaesthesia methods, preoperative VAS scores and dominant hand (Table 4). Anaesthesia methods and follow-up time could predict the cost (Table 4). However, the regression analysis demonstrated that the anaesthesia method was the most powerful predictor for both operating time and cost. We also noted that there was a correlation between preoperative VAS, DASH and Mayo scores because they shared some same items in questionnaires. So did postoperative VAS, DASH, Mayo scores and residual pain.

**Table 2:** Clinical Results and Cost-effectiveness

	GA (***N*** = 51)	WALANT (***N*** = 23)	***p*** Value
Operating time (minutes)	121.5 ± 25.75	88.7 ± 24.51	< 0.001
Coexisting pathology	45(88.2%)	19(82.6%)	0.490
Postop VAS	0 (0 ~ 3)	0 (0 ~ 3)	0.194
Postop Mayo	90 (65 ~ 100)	90 (65 ~ 100)	0.946
Postop DASH	2.3 (0 ~ 25)	2.3 (0 ~ 27.3)	0.424
Residual pain	6 (11.8%)	2 (8.7%)	1.000
Complications	10 (19.6%)	2 (8.7%)	0.320
Recurrence	4 (7.8%)	1 (4.3%)	1.000
Cost (€)	878.7 ± 182.13	487.4 ± 89.15	< 0.001

The cost and the operating time were analyzed using an independent t-test. Fisher’s tests were used to calculate the difference in the rates of coexisting pathology, residual pain, complications, and recurrence. The postop VAS, Mayo, and DASH scores were analyzed using Mann-Whitney U tests

*GA* General anesthesia technique, *WALANT* Wide-awake local anesthesia no tourniquet technique, *VAS* Visual analog scale, *Mayo* Modified Mayo wrist scores, *DASH* Disabilities of Arm, Shoulder and Hand Outcome Measure; €, euro

**Table 3:** The breakdown of the total costs

	Surgical fee	Anaesthesia fee	Admission fee	Total Costs
GA(N = 51)	499 ± 83.33	174.3 ± 17.77	205.4 ± 90.61	878.7 ± 182.13
WALANT(N = 23)	487.4 ± 89.15	0	0	487.4 ± 89.15

*GA* General anesthesia technique, *WALANT* Wide-awake local anesthesia no tourniquet technique

**Table 4:** Results of Regression Analysis for Operating Time and Cost

Parameter	SC	95%CI	***p*** Value
Operating time			
Anaesthesia	−0.467	− 41.74 ~ −17.30	0.000
Postop VAS	0.305	2.18 ~ 10.44	0.003
Dominant	0.198	0.12 ~ 23.48	0.048
Cost			
Anaesthesia	−0.767	−15,933.07 ~ −11,097.58	0.000
Follow-up	−0.308	− 180.59 ~ −69.36	0.000

*SC* Standardised coefficients, *95% CI* 95% confidence interval, *VAS* Visual analog scale

## Discussion

This study demonstrated a significant reduction in total costs and a significant improvement in surgery efficiency among cases performed under WALANT. Furthermore, we found that arthroscopic ganglionectomy under WALANT achieved comparable clinical outcomes to those performed under GA in recurrence rates, residual pain, and functional results.

Theoretically, arthroscopic resection has the advantages of a smaller incision with less damage of surrounding structures, direct visualization of the ganglion stalk, the ability to simultaneously treat coexisting intra-articular pathologies, and rapid functional recoveries. Not surprisingly, arthroscopic treatment for this condition has become state-of-the-art during the last two decades [4]. However, no previous clinical studies have confirmed that arthroscopic resection is superior to open resection [14, 16]. The majority of arthroscopic ganglionectomies have been performed under GA or regional blocks with tourniquets [12, 14]. Because local anaesthesia saves both time and operating room personnel, open excision seemed to be superior to arthroscopic resection of wrist ganglions in time efficiency and cost effectiveness. In recent decades, one of the more noteworthy changes in hand surgery is the method of anaesthesia delivery, specifically the development of WALANT, which was introduced by Dr. Lalonde et al. [5, 7]. Epinephrine hemostasis has obviated the need for tourniquets in most hand surgeries. Removing the need for tourniquets meant that sedation would no longer be required in most of these cases. Patients were able to go home right after surgery, without a need for postoperative medication recovery [10]. Therefore, there are many cost savings associated with this technique, since the anesthesiologist fees, recovery room staff costs, and preoperative testing costs are removed [6]. In addition, the patient under WALANT spent less time in the operating room, which made arthroscopic ganglionectomy more efficient in this study. The most important finding of this investigation was that arthroscopic ganglionectomy under WALANT was superior to that under GA in cost-effectiveness.

Most patients sought treatment for ganglions with a desire to excise the lump. Factors related to ganglion recurrence were more extensive than just a residual stalk [15, 17]. The technical requirements for a successful arthroscopic ganglionectomy are yet to be defined. According to the literature, most studies reported an incidence of ganglion recurrence following arthroscopic excision between 0 and 7% [14]. In this study, the recurrence rate in the WALANT and the GA groups were 4.3 and 7.8%, respectively. Those were compatible to the reported rates. Herein, we found no significant differences between the groups in recurrence rate. Furthermore, regarding functional outcomes, residual pain, or complications, there also were no differences between the two anaesthesia approaches. Thus, we demonstrated that arthroscopic ganglionectomies performed under WALANT was as effective as those performed under GA.

In this study, the complication rates in the WALANT and the GA groups were 8.7 and 19.6%, respectively. The incidence of wrist arthroscopy complications is yet to be thoroughly investigated [18]. The establishment of portals and introduction of the instruments require a thorough knowledge of the wrist anatomy, as well as appropriate skills of the surgeon. Forceful insertion of instruments and poor positioning of the portals can damage neurovascular structures, tendons, ligaments, and articular cartilage [18]. In this study, we found one episode of extensor tendon injury in the GA group. Four cases of extensor tenosynovitis occurred after the surgeries. Not surprisingly, extensor tendon injury could be caused by overly aggressive arthroscopic resections. Several techniques have been recommended to avoid extensor tendon injury. One is using the 6-R portal as visualization portal and then extensor tendons could be seen more clearly during dorsal capsulectomy [4]. Another is a “railroading it” technique, that the extensor tendons were separated from dorsal capsule by using nylon tape while performing a dorsal capsulectomy [19]. Wu and his colleagues [13] recommended the performance of arthroscopic ganglionectomy under local anaesthesia because the patients could feel the pain associated with shaving the tissue outside the capsule while awake. The function of extensor tendon could be evaluated immediately after surgery. In addition, the surgeon would be more likely to set up the portal and insert the instruments gently in awake patients.

Not surprisingly, the concerns related to the safety of epinephrine arose while injecting in finger and hand surgeries. The idea that epinephrine should never be used in finger surgery originated between 1920 and 1940, when procaine was injected with and without epinephrine, with resulting episodes of finger necrosis. Procaine led to necrotic fingers at very acidic pHs, and epinephrine took the blame [7]. Nonetheless, an extensive review of the literature from 1880 to 2000 revealed no documented cases of finger necrosis resulting from local anesthesia with lidocaine plus epinephrine [20]. Recently, Mann and Hammert [21] summarized a large volume of clinical evidence that demonstrated the safety of lidocaine mixed with epinephrine. Another safety concern related to epinephrine is based on its cardiac effects. Some surgeons have reported that the lower concentration of 1/4% lidocaine with 1:400000 epinephrine is effective in the hand if there are concerns for cardiac issues [10]. Generally speaking, ganglions occur predominantly in young women. Therefore, heart health was not a critical issue in our study. There are two minor adverse events that are common following epinephrine injections [7]. One is jitters or shakes. Before injection, surgeons should inform patients that they may feel slightly jittery or shaky following the injection, and that this sensation usually dissipates within 20 min [7, 22]. The other adverse event, vasovagal fainting, can occur following any injection or procedure. Lowering the head and flexing the hips and knees to increase cerebral blood flow is the best management for fainting [23]. In this study, we did not observe any of these adverse events related to epinephrine injection.

Several limitations restrict the scope of this comparison investigation. The first limitation is its retrospective design. Second, our number of patients in the WALANT group was small, although wrist ganglions are not rare. The reason is that the patients were hesitant to undergo arthroscopic ganglionectomy in an awake state. The grouping in this study was based on patients’ preference. Therefore, some selection bias exists. Even though both groups were matched, regarding the demographics and the preoperative clinical assessment, the small sample size would limit the power of a study. Thirdly, we did not record the measurement of patient reported outcomes during the anaesthesia and operation. Therefore, there is insufficient information to evaluate the patient experience or satisfaction of the anaesthesia and operation. Another limitation of the current investigation is the definition of efficiency and cost-effectiveness. With healthcare costs rising worldwide, there has been a greater drive in improving efficiency, but the definition of efficiency and cost-effectiveness has been nebulous. There is a need to design an objective system to evaluate them. To determine the difference in efficiency and cost-effectiveness between the WALANT and GA groups, we reviewed the operating times and the total costs and then we found a significant reduction in total costs and a significant improvement in surgery efficiency among cases performed under WALANT.

## Conclusions

While undergoing arthroscopic excision of wrist ganglions, both anesthesia techniques were effective and safe with respect to recurrence, complications, and residual pain rates. However, no extensor tenosynovitis or tendon injury was observed under the WALANT technique. The most important finding of this study was that arthroscopic ganglionectomy under WALANT was superior to that under GA in cost-effectiveness.

## Supplementary information

**Additional file 1.**

## Data Availability

The datasets used and/or analysed during the current study are available from the corresponding author on reasonable request.

## Abbreviations

DASH: Disabilities of Arm, Shoulder and Hand (score)
GA: General anaesthesia
LT: Lunotriquetral (ligament)
MRI: Magnetic resonance image
SL: Scapholunate (ligament)
TFCC: Triangular fibrocartilage complex
VAS: Visual analog scale
WALANT: Wide-awake local anaesthesia no tourniquet
