# Supplementary figures and images for: A cost and efficacy analysis of performing arthroscopic excision of wrist ganglions under wide-awake anaesthesia versus general anaesthesia
Source: BMC Musculoskelet Disord. 2020 Jul 13;21:459. doi: 10.1186/s12891-020-03482-0 (PMC7359493; doi:10.1186/s12891-020-03482-0)

Document 1


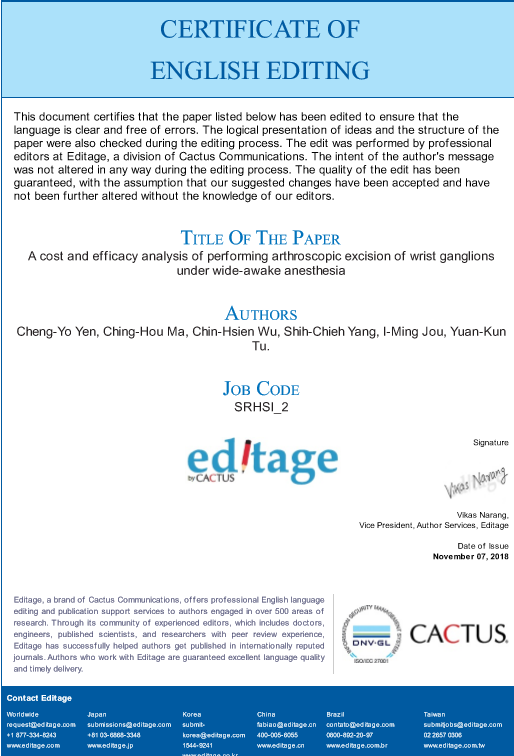


Document 2


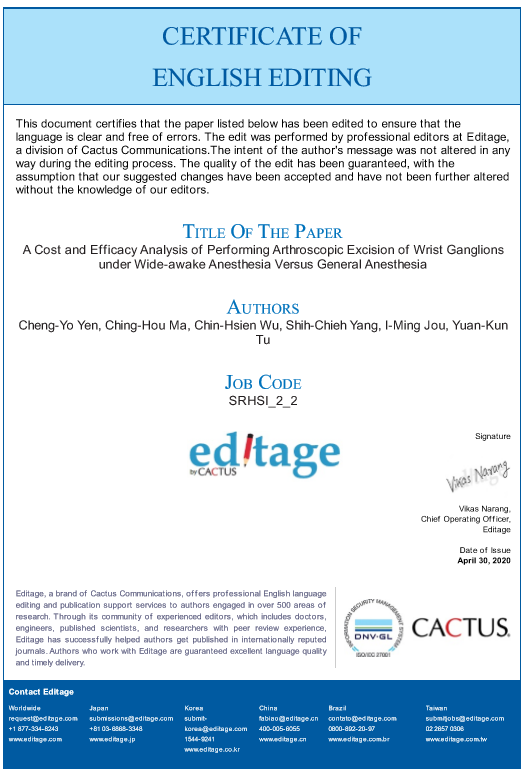


Document 3


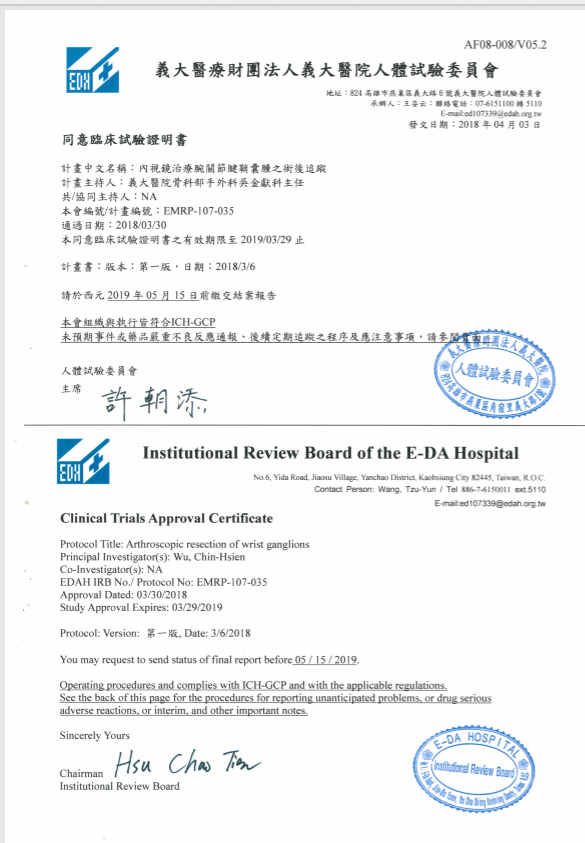

Supplement: Supplementary file 1 — Additional file 1. [file 12891_2020_3482_MOESM1_ESM.docx]
